# Role of left ventricular strain measurements and native T1 and T2 mapping on cardiac magnetic resonance imaging in evaluating early left ventricle myocardial derangement in patients with high normal blood pressure

**DOI:** 10.34172/jcvtr.026.33467

**Published:** 2026-03-30

**Authors:** Rishabh Khurana, Priya Jagia, Vineeta Ojha, Sanjeev Kumar, Ambuj Roy, Maroof Ahmed Khan, Niraj Nirmal Pandey

**Affiliations:** ^1^Department of Cardiovascular Radiology & Endovascular Interventions, All India Institute of Medical Sciences, New Delhi-110029, India; ^2^Department of Cardiology, All India Institute of Medical Sciences, New Delhi-110029, India; ^3^Department of Biostatistics, All India Institute of Medical Sciences, New Delhi-110029, India

**Keywords:** Global longitudinal strain, Heart ventricles, Blood pressure, Magnetic resonance imaging

## Abstract

**Introduction:**

The study sought to evaluate changes in left ventricular (LV) strain and native T1/T2 mapping characteristics on cardiac magnetic resonance imaging (CMR) in patients with high-normal blood pressure (HNBP).

**Methods:**

A prospective case-control study including 25 cases having HNBP and 25 age- and sex-matched healthy controls was conducted. LV strain was evaluated on CMR using feature tracking and 2-dimensional and 3-dimensional longitudinal, circumferential and radial strain values were calculated. Native T1/T2 mapping values were also calculated.

**Results:**

Subclinical impairment of LV mechanics was evident in the form of deranged LV strain parameters in cases with HNBP compared to controls. The two-dimensional global radial (25.34±3.06 vs. 28.52±5.69; *P*=0.0323), global circumferential (-16.05±1.31 vs. -17.27±2.23; *P*=0.0241) and global longitudinal strain (-16.33±2.24 vs. -16.49±7.25); *P*=0.0193) and three-dimensional global circumferential strain (-13.94±10.81 vs. -17.84±2.78; *P*=0.0133) values were significantly impaired in cases compared to controls. No significant difference was observed in the native T1/T2 mapping parameters.

**Conclusion:**

LV strain parameters are significantly deranged in patients with HNBP, compared to healthy controls, in the absence of other morphological changes or interstitial fibrosis. Impaired LV strain parameters can serve as a new marker for detection of subclinical myocardial dysfunction in patients with HNBP having preserved chamber function.

## Introduction

 Hypertension (HT) is a major risk factor for development of cardiovascular (CV) disease and cerebrovascular accidents (CVA). As per the 2018 European Society of Cardiology (ESC)/ European Society of Hypertension (ESH) guidelines, HT is defined as systolic blood pressure (SBP) > 140 mm Hg and/or diastolic blood pressure (DBP) > 90 mm Hg. Patients having “high normal blood pressure” [HNBP; SBP (130-139 mm Hg) and/or DBP (85-89 mm Hg), not taking any antihypertensive medications] are deemed to be at risk for developing hypertension.^1^ In earlier classifications, this range of blood pressure was classified under the category of “prehypertension” (Pre-HT). Pre-HT is also associated with excessive CV morbidity and augments the risk of coronary artery disease (CAD), CVA as well as heart failure (HF), independent of other CV risk factors.^2^

 Studies have shown development of left ventricular diastolic dysfunction (LVDD) in not only hypertensive individuals, but also in patients with Pre-HT.^3^ Moreover, there is alteration in the left ventricular (LV) mechanical properties as evident by derangement of LV strain.^4,5^ Due to its high spatial and temporal resolution, cardiac magnetic resonance (CMR) imaging has become the gold standard for assessment of LV function and strain analysis.^6^ Also, a strong correlation between myocardial native T1 values and histological myocardial fibrosis has been observed.^7^

 The present study sought to evaluate whether patients with HNBP had significant changes in LV structure and function compared to healthy controls, by assessing LV strain as well as analysing T1 and T2 mapping characteristics on CMR, thereby identifying possible early markers of hypertension-mediated organ damage (HMOD).

## Materials and Methods

 A prospective case-control study including 50 subjects (25 cases and 25 controls) was performed. Office blood pressure (BP) measurements were obtained in the sitting position according to the standard protocol.^1^ Cases comprised of subjects (age > 16 years) having SBP: 130-139 and/or DBP: 85-89 mm Hg, in the absence of any associated comorbidities. The control group comprised of healthy subjects (SBP: < 120 and DBP: < 80 mm Hg). Cases and controls were matched for age ( ± 2 years) and sex. Exclusion criteria included a previous diagnosis of hypertension, history of treatment with anti-hypertensive drugs, diabetes mellitus or history of treatment with antidiabetic drugs, ECG-stress test positive for CAD, impaired LV systolic function, cardiomyopathy, HF or any valvular heart disease. The study protocol was approved by the institutional ethics committee and all study participants provided written informed consent.

###  CMR technique 

 CMR imaging was performed using a 1.5 Tesla MRI scanner (MAGNETOM Aera, Siemens Healthineers, Erlangen, Germany) using a cardiac coil. The CMR protocol is described in Appendix 1.

 Two experienced observers (having > 5 years’ experience in CMR) independently evaluated all CMR scans. LV functional analysis was performed using Circle Cvi42 (Cardiovascular Imaging Inc., Calgary, Alberta, Canada)]. Native T1 and T2 mapping values were estimated after tracing a ROI in the mid interventricular septum, and mean values were reported. LV strain was evaluated using the Feature Tracking (FT) post processing tool. Longitudinal, circumferential and radial strains (global, segmental and according to AHA segmentation model in Bull’s eye plot) were estimated. Both 2D and 3D strain values were evaluated. Global radial strain (GRS) and global circumferential strain (GCS) were derived from the short axis images, whereas the global longitudinal strain (GLS) was derived from the 4-chamber, 2-chamber and 3-chamber images. Additional radial, circumferential and longitudinal strain (RS, CS and LS respectively) parameters were derived at basal, mid ventricular and apical levels of LV. The methodology of LV strain evaluation has been depicted in Supplementary file, Figure S1.

**Figure 1:**
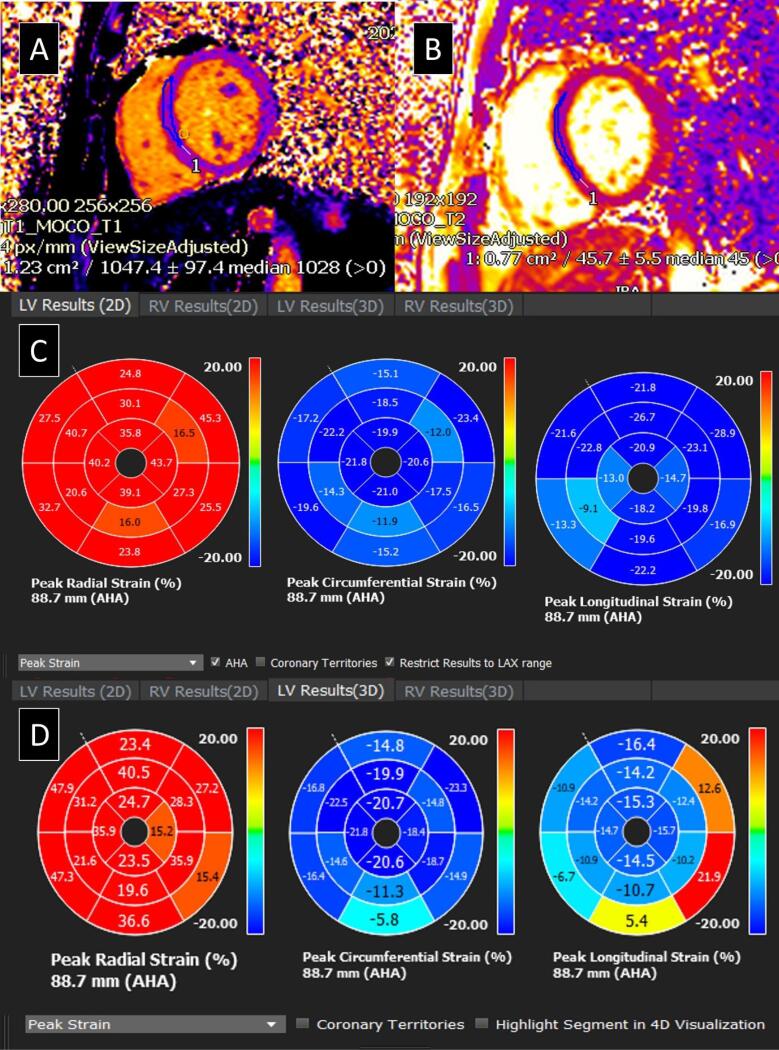
Left Ventricular Strain and Mapping analysis of a healthy normal control subject (A and B) Native T1 and T2 mapping performed on T1 and T2 mapping module of Circle Cvi42 (Cardiovascular Imaging Inc., Calgary, Alberta, Canada). (C and D) Two-dimensional (2-D) global and segmental, circumferential, radial and longitudinal, strain; and Three-dimensional (3-D) global and segmental, circumferential, radial and longitudinal, strain; derived on Circle Cvi42 (Cardiovascular Imaging Inc., Calgary, Alberta, Canada), using the post processing tool - Feature Tracking (FT-CMR) software. The two-dimensional global radial, global circumferential and global longitudinal strain values (in %) were 28.58, -17.51 and -18.68 respectively while the three-dimensional global radial, global circumferential and global longitudinal strain values (in %) were 27.02, -17.87 and -12.28 respectively

**Figure 2:**
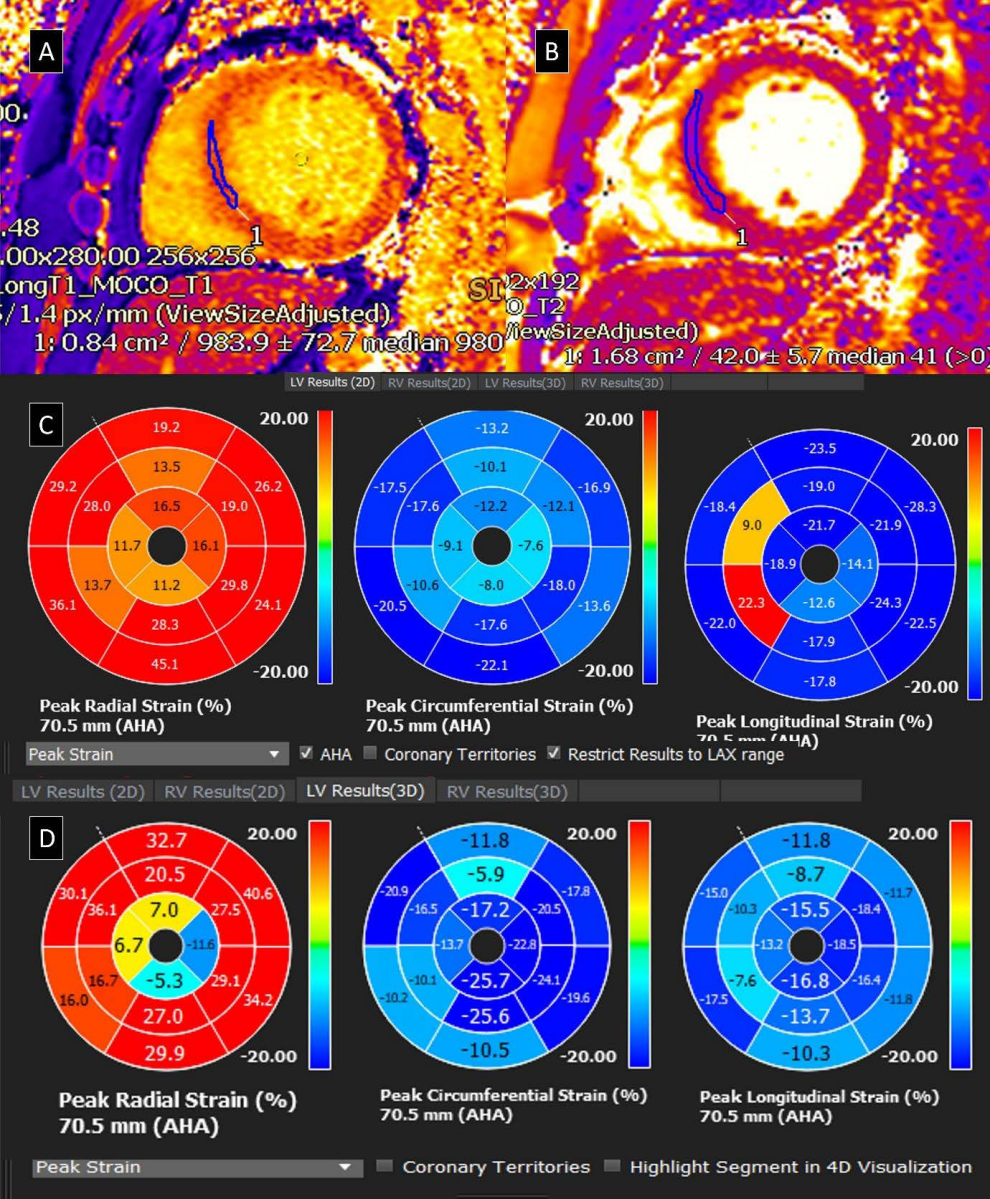
Left Ventricular Strain & Mapping analysis of a patient with high normal blood pressure (A and B) Native T1 and T2 mapping performed on T1 and T2 mapping module of Circle Cvi42 (Cardiovascular Imaging Inc., Calgary, Alberta, Canada). (C and D) Two-dimensional (2-D) global and segmental, circumferential, radial and longitudinal, strain; and Three-dimensional (3-D) global and segmental, circumferential, radial and longitudinal, strain; derived on Circle Cvi42 (Cardiovascular Imaging Inc., Calgary, Alberta, Canada), using the post processing tool - Feature Tracking (FT-CMR) software. The two-dimensional global radial, global circumferential and global longitudinal strain values (in %) were 20.28, -13.55 and -16.95 respectively while the three-dimensional global radial, global circumferential and global longitudinal strain values (in %) were 15.49, -17.29 and -13.88 respectively

Figures 1 and 2 depict the global and segmental strain parameters, derived on CMR, along with native T1 and T2 mapping assessment, in a representative subject from each group.

###  Statistical analysis

 All statistical calculations were performed using Statistical Package for Social Sciences (SPSS) version 23.0. Continuous variables have been presented as means with standard deviations or as medians with interquartile ranges, depending on normality of distribution and compared using Student’s t test and Kruskal Wallis test (along with Mann-Whitney U test) respectively. Categorical variables were expressed as frequencies with percentages and compared using Chi-squared test or Fisher’s exact test as appropriate. Correlation coefficient was estimated to assess the relation between variables. A receiver operating characteristics (ROC) analysis was also performed. For all statistical tests, a p value < 0.05 was considered significant.

## Results

 The demographic details of the study participants have been depicted in Table 1. No significant difference was observed in the baseline demographic parameters.

**Table 1:** Demographic details of study population

**Parameters**	**Group**		***P*** ** value**
	**Case (n=25)**	**Control (n=25)**	
Age (Years) Mean ± SD	34.56 ± 8.61	34.52 ± 8.58	0.987^1^
Gender			1.000^2^
Male	21 (84.0%)	21 (84.0%)	
Female	4 (16.0%)	4 (16.0%)	
Weight (kgs)	71.36 ± 12.40	73.48 ± 7.85	0.474^1^
Height (cms)	167.88 ± 12.07	171.68 ± 8.11	0.199^1^
BMI (kg/m^2^)	25.41 ± 4.38	24.99 ± 2.77	0.574^3^
SBP Average (mm Hg)***	131.20 ± 6.66	116.24 ± 2.17	< 0.001^3^
DBP Average (mm Hg)***	83.64 ± 4.43	73.48 ± 2.37	< 0.001^3^

***Significant at *P* < 0.05, 1: *t*-test, 2: Fisher’s Exact Test, 3: Wilcoxon-Mann-Whitney U Test

###  Comparative Assessment of Left Ventricular Morphological, Functional, Strain and Mapping Parameters in Cases and Controls in study population

 The comparative assessment of left ventricular morphological, functional, strain and mapping parameters between the two groups, have been summarised in Table 2. The ROC curve analysis, showing diagnostic performances, of various parameters in differentiating case and control groups, have been depicted in Supplementary File, Table S1. The cut-offs of different variables in the comparative assessment (i.e., Table 2), for predicting the cases and controls, was estimated. Their sensitivity, specificity, positive predictive value (PPV) and negative predictive value (NPV), diagnostic accuracy (DA) and odds ratio (OR) have been depicted in Supplementary File, Table S2.

**Table 2:** Summary of comparative Assessment of Left Ventricular Morphological, Functional, Strain and Mapping Parameters in Cases & Controls

**Parameters**	**Group**		***P*** ** value**
	**Case (n=25)**	**Control (n=25)**	
Left ventricular Ejection fraction (LVEF) MRI	61.71 ± 5.69	62.41 ± 4.73	0.286^3^
Left Ventricular End Diastolic Volume	130.99 ± 28.42	137.15 ± 22.08	0.396^1^
Left Ventricular End Systolic Volume	51.13 ± 14.37	53.25 ± 10.37	0.553^1^
Left Ventricular Stroke Volume	80.79 ± 18.12	85.43 ± 14.36	0.321^1^
Left ventricle End Diastolic Volume Indexed (LVEDVi)	72.12 ± 12.17	74.24 ± 11.68	0.393^3^
Left ventricle End systolic Volume Indexed (LVESVi)	28.16 ± 6.80	27.92 ± 5.46	0.893^1^
LV Stroke Volume Indexed (LVSVi)	44.13 ± 7.73	47.39 ± 9.13	0.179^1^
LV Myocardial Indexed Mass	46.48 ± 8.76	49.91 ± 18.36	0.954^3^
LV Myocardial Total Mass	81.04 ± 18.25	77.73 ± 20.36	0.548^1^
LV Radial Strain: Global - MRI 2D***	25.34 ± 3.06	28.52 ± 5.69	0.032^3^
LV Radial Strain: Basal - MRI 2D	25.00 ± 3.91	26.96 ± 3.44	0.067^1^
LV Radial Strain: Mid - MRI 2D***	23.17 ± 3.53	26.14 ± 5.91	0.037^1^
LV Radial Strain: Apical - MRI 2D***	32.19 ± 8.85	39.60 ± 12.64	0.028^3^
LV Circumferential Strain: Global- MRI 2D***	-16.05 ± 1.31	-17.27 ± 2.23	0.024^1^
LV Circumferential Strain: Basal- MRI 2D	-15.80 ± 1.66	-16.59 ± 1.50	0.085^1^
LV Circumferential Strain: Mid - MRI 2D***	-15.19 ± 1.65	-16.52 ± 2.54	0.034^1^
LV Circumferential Strain: Apical- MRI 2D***	-18.68 ± 3.30	-20.75 ± 3.58	0.039^1^
LV longitudinal Strain: Global - MRI 2D***	-16.33 ± 2.24	-16.49 ± 7.25	0.019^3^
LV longitudinal Strain: 4 chamber - MRI 2D***	-15.40 ± 2.67	-17.21 ± 2.25	0.012^1^
LV longitudinal Strain: 2 Chamber VLA - MRI 2D	-16.63 ± 3.33	-18.17 ± 3.29	0.105^1^
LV longitudinal Strain: 3 Chamber LVO- MRI 2D	-17.04 ± 2.81	-18.41 ± 2.21	0.062^1^
LV Radial Strain: Global - MRI - 3D	33.50 ± 22.61	32.73 ± 9.39	0.347^3^
LV Radial Strain: Basal - MRI 3D	36.72 ± 7.87	41.42 ± 9.33	0.060^1^
LV Radial Strain: Mid - MRI 3D	30.63 ± 20.86	31.07 ± 14.51	0.574^3^
LV Radial Strain: Apical - MRI 3D	53.71 ± 118.50	33.05 ± 23.20	0.832^3^
LV Circumferential Strain: Global- MRI 3D***	-13.94 ± 10.81	-17.84 ± 2.78	0.013^3^
LV Circumferential Strain: Basal- MRI 3D	-14.88 ± 2.45	-15.35 ± 2.01	0.469^1^
LV Circumferential Strain: Mid - MRI 3D***	-16.19 ± 1.78	-17.73 ± 3.21	0.043^1^
LV Circumferential Strain: Apical- MRI 3D	-18.07 ± 3.62	-20.38 ± 3.86	0.059^3^
LV longitudinal Strain: Global - MRI 3D	-12.40 ± 3.41	-13.57 ± 2.32	0.163^1^
LV longitudinal Strain: Basal - MRI 3D	-9.66 ± 4.52	-11.17 ± 2.81	0.164^1^
LV longitudinal Strain: Mid - MRI 3D	-12.27 ± 4.24	-13.46 ± 2.17	0.218^1^
LV longitudinal Strain: Apical - MRI 3D	-15.08 ± 3.11	-16.09 ± 1.71	0.165^1^
T1 Mapping	1005.20 ± 30.53	1014.16 ± 31.31	0.265^3^
T2 Mapping	48.17 ± 3.28	47.46 ± 2.50	0.390^1^

***Significant at *P* < 0.05, 1: *t*-test, 2: Fisher’s Exact Test, 3: Wilcoxon-Mann-Whitney U Test

## Discussion

 Based upon results of various clinical trials, various guidelines have been laid down for the diagnosis of HT, and cut-off BP values have been defined, which are sharp and narrow. Of late, several studies have demonstrated that there exists a continuous relationship between BP and various CV events and CVA. This makes a line of demarcation (cut-off values) between normotension and HT, a gray zone.^8,9^ This is substantiated by the fact that various epidemiological associations between BP and CV risk extend from very low levels of BP onwards (i.e., SBP > 115 mmHg) with increase in developing the risk for several ailments in various organ systems of the body leading to target organ damage in the form of alteration in the morphology with or without change in functions.^9^ A meta-analysis demonstrated that a BP of 120-129 mmHg systolic and 80-84 mmHg diastolic was associated with a hazard ratio of 1.1 to 1.5 for CV events, whereas BP of 130-139 mmHg systolic and 85-89 mmHg diastolic was associated with a hazard ratio of 1.5 to 2.0.^8,10-12^ The classification of BP (and terminology of its categories) also varies across different societies across the globe.^10-13^

 In Pre-HT, BP remains a strong predictor of CV events even after a statistical adjustment for other risk factors, which suggests that a reduction of BP might be beneficial.^14^ Therefore, it has been emphasised that the BP categorised as “Prehypertension”, “High Normal Blood Pressure” or “Elevated Blood Pressure”, according to definitions requires attention, intervention and health-promoting lifestyle modifications even at an earlier stage to prevent subsequent progressive rise in BP and development of CV events.^1,10^

 Systemic arterial hypertension leads to alteration of cardiac micro-environment in the form of cardiomyocyte hypertrophy, arteriolar hypertrophy, endothelial damage, neovascular stimulation and development of interstitial changes (connective tissue hyperplasia, induction of collagen production by fibroblasts and subsequent development of different types of fibrosis including reactive interstitial fibrosis, infiltrative interstitial fibrosis and replacement fibrosis. The reactive type of fibrosis may regress, while in the replacement type, loss of myocyte is permanent due to necrosis and/or apoptosis).^15^ Changes in morphology and function of the heart due to chronically elevated LV afterload in patients with systemic hypertension can be seen as LV hypertrophy (LVH), LA enlargement, elevated risk of arrythmias and Heart Failure.^1^ It has been observed that Pre-HT patients show evidence of precocious HMOD in the form of reactive interstitial response, LV structural and functional changes which can impact diastolic or systolic LV function.^4,5,16^

###  Cardiac morphological changes in patients with High Normal Blood Pressure in comparison to Normotensive subjects

 There was no evidence of LVH or chamber dilatation either in HNBP group or the control group in our study. No significant difference was seen between LV mass (LVM), end-diastolic volume (EDV) and end-systiolic volume (ESV) between cases and controls. Our results are in conformity with the study by Escudero et al in which no evidence of LVH was observed in either of the group. Nevertheless, they found that indexed LV mass (LVMi) was higher in HNBP group in comparison to normotensive control group (although the values were not high enough to suggest LVH).^17^ On the contrary, various studies available in literature revealed presence of higher LVM, LVMi, LV wall thickness (LVWT) or higher prevalence of LVH, in Pre-HT subjects in comparison to the NT counterparts.^16,18,19^ Similarly, a higher LVMi and LVWT along with greater prevalence of abnormal LV geometry was elucidated in subjects with HT and Pre-HT in comparison to NT controls by Santos et al.^3^

###  Functional changes in Heart of patients with High Normal Blood Pressure in comparison to Normotensive subjects

 There were no significant differences between LV ejection fraction, EDV (or indexed EDV), ESV (or indexed ESV), stroke volume (SV) (or indexed SV) in our study. The diagnostic performance of various strain parameters (global and segmental), on CMR, has been reflected in the results section. Our study shows the existence of subclinical impairment of LV mechanics in the form of deranged LV strain parameters in HNBP subjects in comparison to age- and sex- matched normotensive controls, which is in lines with the results of Tadic et al.^4^

###  Mapping of LV Myocardium of patients with High Normal Blood Pressure in comparison to Normotensives subjects

 In our study, mapping (both Native T1 and T2) analysis did not reveal any significant difference between patients with HNBP in comparison to controls. Intravenous Gadolinium was not administered. Hence, post contrast T1 mapping to estimate extracellular volume (ECV) could not be performed. Kuruvilla et al performed CMR using 1.5T to evaluate native T1 mapping and mean ECV in subjects having HT with LVH, HT without LVH and compared them with NT controls.^20^ Higher native T1 mapping values were observed in patients having HT with LVH (in comparison to NT controls); and higher ECV values were seen in patients with HT with LVH (in comparison to patients having HT without LVH and NT controls) suggesting that native T1 mapping and ECV measurement can provide a non-invasive assessment of diffuse fibrosis in hypertensive heart disease.^20^ In the present study, although the mapping values did not reflect any significant differences between the two groups, the global strain parameters revealed a significant difference between LV mechanics of HNBP and NT subjects. So, strain abnormality may possibly predate development of interstitial fibrosis. This may possibly indicate that alterations in LV mechanics occur even before the development of interstitial fibrosis in patients with HNBP.

 Several limitations need to be acknowledged. Firstly, ambulatory blood pressure monitoring was not performed due to technical issues due to Covid-19 pandemic during the study. Secondly, a matched cohort of hypertensive patients were not included. Thirdly, the study was a single centre prospective study. The generalizability of these findings across various age and ethnic/racial groups may be limited. Moreover, the causal relationship between HNBP and LV strain deformation cannot be established. This needs to be elucidated in further multicentric studies, including a variety of ethnic/racial groups. Additionally, the effect of adopting lifestyle modification, and possible antihypertensive treatment, to demonstrate the extent of any degree of reversibility in altered LV mechanics can be evaluated in future experimental studies.

## Conclusion

 This study demonstrates the presence of early functional abnormalities in the form of deranged strain parameters, using CMR, in participants with HNBP. This altered LV mechanics may precede development of other morphological changes and development of different stages of fibrosis in HNBP patients. Strain imaging has the potential to serve as a new marker for subclinical myocardial dysfunction in patients with HNBP having preserved chamber function.

## Competing Interests

 None.

## Ethical Approval

 The study was carried out after approval of Institutional Ethics Committee (IECPG-481/17.07.2019, RT-03/26.09.2019). All procedures were in accordance with the ethical standards of the institutional and/or national research committee and with the 1964 Helsinki declaration and its later amendments or comparable ethical standards.

## Supplementary Files

Supplementary file 1 contains Table S1-S2 and Figure S1.

## Appendix

### Appendix 1

**CMR protocol:**

**The following sequences were acquired in all cases:**

(a) Localizers
(b) white blood TruFISP (true fast imaging with steady-state free precession) coronal and axial stack
(c) ECG- and respiratory-gated balanced steady-state free precession (SSFP) - short axis cine images - 6 to 8 mm, 25 phases
(d) ECG- and respiratory-gated balanced SSFP - four chamber cine images - 6 to 8 mm, 25 phases
(e) ECG- and respiratory-gated balanced SSFP - two chamber cine images - 6 to 8 mm, 25 phases
(f) ECG- and respiratory-gated balanced SSFP - one slice of 3 chamber cine image (for assessment of left ventricular outflow)
(g) Native T1 map [Modified Look-Locker Inversion Recovery (MOLLI)] - short axis three slices at basal, mid and apical LV (according to Society for Cardiovascular Magnetic Resonance [SCMR] guidelines)
(h) T2 map (T2 prepared single-shot SSFP) - short axis three slices each at basal, mid and apical LV (according to SCMR guidelines); (i) T2 star map- one short axis slice (according to SCMR guidelines).
