# Supplementary material for: Role of left ventricular strain measurements and native T1 and T2 mapping on cardiac magnetic resonance imaging in evaluating early left ventricle myocardial derangement in patients with high normal blood pressure
Source: J Cardiovasc Thorac Res. 2026 Mar 30;18(1):55–61. doi: 10.34172/jcvtr.026.33467 (PMC13309326; doi:10.34172/jcvtr.026.33467)
Supplement: Supplementary file 1 — contains Table S1-S2 and Figure S1. [file jcvtr-18-55-s001.pdf]

J Cardiovasc Thorac Res. 2026;18(1):55-S1
doi:10.34172/jcvtr.026.33467
http://jcvtr.tbzmed.ac.ir

Table S1. Comparison of the Diagnostic Performance of Various Parameters in Predicting Case vs Control

Predictor	AUROC	95% CI	P-value	Sn	Sp	PPV	NPV	DA
Left ventricle Ejection fraction (LVEF) MRI	0.589	0.423-0.754	0.286	68%	60%	63%	65%	64%
Left Ventricular End Diastolic Volume	0.613	0.448-0.777	0.174	56%	76%	70%	63%	66%
Left Ventricular End Systolic Volume	0.566	0.401-0.732	0.426	56%	68%	64%	61%	62%
Left Ventricular Stroke Volume	0.592	0.428-0.756	0.269	60%	72%	68%	64%	66%
Left ventricle End Diastolic Volume Indexed (LVEDVi)	0.571	0.407-0.735	0.393	64%	60%	62%	62%	62%
Left ventricle End systolic Volume Indexed (LVESVi)	0.504	0.337-0.671	0.969	36%	80%	64%	56%	58%
LV Stroke Volume Indexed (LVSVi)	0.597	0.436-0.758	0.244	72%	52%	60%	65%	62%
LV Myocardial Indexed Mass	0.522	0.355-0.688	0.801	64%	60%	62%	62%	62%
LV Myocardial Total Mass	0.526	0.362-0.691	0.756	28%	88%	70%	55%	58%
LV Radial Strain: Global - MRI 2D	0.677	0.525-0.829	**0.033**	64%	68%	67%	65%	66%
LV Radial Strain: Basal - MRI 2D	0.677	0.522-0.831	**0.033**	64%	76%	73%	68%	70%
LV Radial Strain: Mid - MRI 2D	0.673	0.519-0.827	**0.037**	64%	68%	67%	65%	66%
LV Radial Strain: Apical - MRI 2D	0.682	0.532-0.832	**0.028**	56%	76%	70%	63%	66%
LV Circumferential Strain: Global- MRI 2D	0.683	0.532-0.834	**0.027**	92%	40%	60%	83%	66%
LV Circumferential Strain: Basal- MRI 2D	0.669	0.513-0.824	**0.042**	64%	72%	70%	67%	68%
LV Circumferential Strain: Mid - MRI 2D	0.687	0.535-0.84	**0.024**	68%	68%	68%	68%	68%
LV Circumferential Strain: Apical- MRI 2D	0.662	0.51-0.815	0.050	80%	48%	61%	71%	64%
LV longitudinal Strain: Global - MRI 2D	0.694	0.544-0.845	**0.019**	72%	68%	69%	71%	70%
LV longitudinal Strain: 4 chamber - MRI 2D	0.722	0.576-0.869	**0.007**	76%	68%	70%	74%	72%
LV longitudinal Strain: 2 Chamber VLA - MRI 2D	0.630	0.472-0.788	0.116	88%	40%	59%	77%	64%
LV longitudinal Strain: 3 Chamber LVO- MRI 2D	0.677	0.526-0.828	**0.033**	60%	72%	68%	64%	66%

LV Radial Strain: Global - MRI - 3D	0.578	0.416-0.741	0.347	68%	52%	59%	62%	60%
LV Radial Strain: Basal - MRI 3D	0.646	0.492-0.801	0.077	68%	56%	61%	64%	62%
LV Radial Strain: Mid - MRI 3D	0.547	0.384-0.711	0.574	92%	24%	55%	75%	58%
LV Radial Strain: Apical - MRI 3D	0.518	0.354-0.683	0.831	28%	84%	64%	54%	56%
LV Circumferential Strain: Global-MRI 3D	0.706	0.56-0.851	**0.013**	80%	60%	67%	75%	70%
LV Circumferential Strain: Basal- MRI 3D	0.572	0.409-0.735	0.388	56%	64%	61%	59%	60%
LV Circumferential Strain: Mid - MRI 3D	0.649	0.49-0.808	0.073	96%	40%	62%	91%	68%
LV Circumferential Strain: Apical- MRI 3D	0.657	0.503-0.811	0.059	92%	36%	59%	82%	64%
LV longitudinal Strain: Global - MRI 3D	0.631	0.469-0.794	0.114	64%	72%	70%	67%	68%
LV longitudinal Strain: Basal - MRI 3D	0.598	0.43-0.766	0.237	48%	84%	75%	62%	66%
LV longitudinal Strain: Mid - MRI 3D	0.621	0.456-0.786	0.146	56%	76%	70%	63%	66%
LV longitudinal Strain: Apical - MRI 3D	0.618	0.457-0.779	0.157	48%	80%	71%	61%	64%
T1 Mapping	0.593	0.426-0.76	0.265	76%	56%	63%	70%	66%
T2 Mapping	0.557	0.393-0.721	0.497	28%	96%	88%	57%	62%

AUROC: Area under ROC curve; CI: Confidence interval; Sn: Sensitivity; Sp: Specificity; PPV: Positive predictive value; NPV: Negative predictive value; DA: Diagnostic Accuracy.

Table S2. Performance of Study Parameters for Predicting Group: Case vs Control

**Variable**	**Cut-off suggesting Outcome Present**	**Sensitivity**	**Specificity**	**Positive predictive value**	**Negative predictive value**	**Diagnostic Accuracy**	**Odds Ratio**	**P Value**
LV Radial Strain: Global - MRI 2D (Cutoff: 25.92 by ROC)	≤25.92	64.0% (43-82)	68.0% (46-85)	66.7% (45-84)	65.4% (44-83)	66.0% (51-79)	3.78 (1.17-12.19)	**0.024**
LV Radial Strain: Mid - MRI 2D (Cutoff: 24.18 by ROC)	≤24.18	64.0% (43-82)	68.0% (46-85)	66.7% (45-84)	65.4% (44-83)	66.0% (51-79)	3.78 (1.17-12.19)	**0.024**
LV Radial Strain: Apical - MRI 2D	≤31.69	56.0% (35-76)	76.0% (55-91)	70.0% (46-88)	63.3% (44-80)	66.0% (51-79)	4.03 (1.20-13.53)	**0.021**

(Cutoff: 31.69 by ROC)								
LV Circumferential Strain: Global- MRI 2D (Cutoff: -17.59 by ROC)	≥-17.59	92.0% (74-99)	40.0% (21-61)	60.5% (43-76)	83.3% (52-98)	66.0% (51-79)	7.67 (1.47-39.99)	**0.008**
LV Circumferential Strain: Mid - MRI 2D (Cutoff: -15.87 by ROC)	≥-15.87	68.0% (46-85)	68.0% (46-85)	68.0% (46-85)	68.0% (46-85)	68.0% (53-80)	4.52 (1.38-14.82)	**0.011**
LV Circumferential Strain: Apical- MRI 2D (Cutoff: -20.67 by ROC)	≥-20.67	80.0% (59-93)	48.0% (28-69)	60.6% (42-77)	70.6% (44-90)	64.0% (49-77)	3.69 (1.05-12.96)	**0.037**
LV longitudinal Strain: Global - MRI 2D (Cutoff: -17.01 by ROC)	≥-17.01	72.0% (51-88)	68.0% (46-85)	69.2% (48-86)	70.8% (49-87)	70.0% (55-82)	5.46 (1.63-18.36)	**0.005**
LV longitudinal Strain: 4 chamber - MRI 2D (Cutoff: -16.13 by ROC)	≥-16.13	76.0% (55-91)	68.0% (46-85)	70.4% (50-86)	73.9% (52-90)	72.0% (58-84)	6.73 (1.94-23.36)	**0.002**
LV Circumferential Strain: Global- MRI 3D (Cutoff: -17.53 by ROC)	≥-17.53	80.0% (59-93)	60.0% (39-79)	66.7% (47-83)	75.0% (51-91)	70.0% (55-82)	6.00 (1.69-21.26)	**0.004**
LV Circumferential Strain: Mid - MRI 3D (Cutoff: -18.4 by ROC)	≥-18.4	96.0% (80-100)	40.0% (21-61)	61.5% (45-77)	90.9% (59-100)	68.0% (53-80)	16.00 (1.86-137.97)	**0.002**

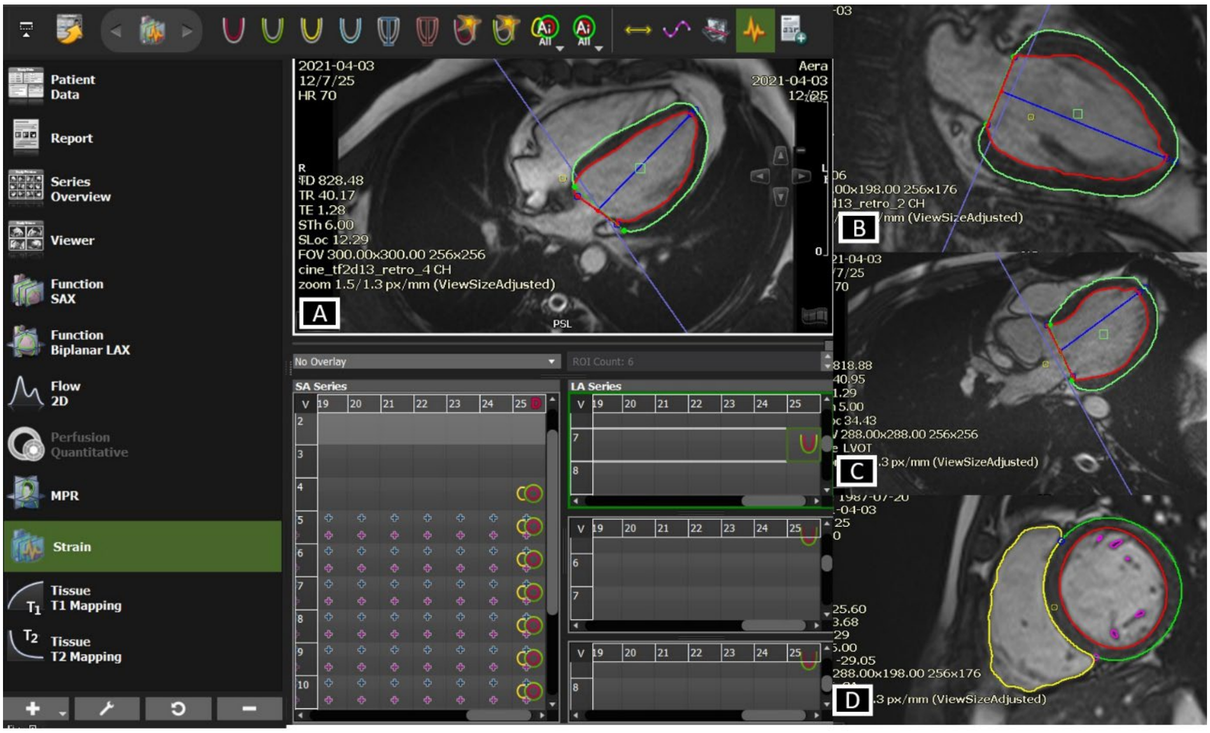

Figure S1. Methodology of strain evaluation, on Circle Cvi42 (Cardiovascular Imaging Inc., Calgary, Alberta, Canada), using the post processing tool - Feature Tracking (FT-CMR)

Left ventricular (LV) endocardial (red) and epicardial (green) contours have been traced on 4-Chamber **(A),** 2-Chamber **(B),** 3-Chamber **(C)** and Short Axis **(D)** views. Global radial strain (GRS) and global circumferential strain (GCS) were derived from the short axis images **(D),** whereas global longitudinal strain (GLS) was derived from the 4-chamber **(A),** 2-chamber **(B)** and 3-chamber images **(C).** Both two-dimensional (2D) and three-dimensional (2D) strain analysis was performed. Segmental strain analysis was also evaluated on basal, mid-ventricular and apical LV segments
